# A Single Nucleotide Polymorphism within *DUSP9* Is Associated with Susceptibility to Type 2 Diabetes in a Japanese Population

**DOI:** 10.1371/journal.pone.0046263

**Published:** 2012-09-27

**Authors:** Hisashi Fukuda, Minako Imamura, Yasushi Tanaka, Minoru Iwata, Hiroshi Hirose, Kohei Kaku, Hiroshi Maegawa, Hirotaka Watada, Kazuyuki Tobe, Atsunori Kashiwagi, Ryuzo Kawamori, Shiro Maeda

**Affiliations:** 1 Laboratory for Endocrinology and Metabolism, RIKEN Center for Genomic Medicine, Yokohama 230-0045, Japan; 2 Department of Internal Medicine, Division of Metabolism and Endocrinology, St. Marianna University School of Medicine, Kawasaki, Kanagawa, Japan; 3 First Department of Internal Medicine, University of Toyama, Toyama, Japan; 4 Health Center, Keio University School of Medicine, Tokyo, Japan; 5 Division of Diabetes, Endocrinology and Metabolism, Department of Internal Medicine, Kawasaki Medical School, Kurashiki, Okayama, Japan; 6 Department of Medicine, Shiga University of Medical Science, Otsu 520-2192, Japan; 7 Department of Medicine, Metabolism and Endocrinology, School of Medicine, Juntendo University, 2-1-1 Hongo, Bunkyo-ku, Tokyo 113-8421, Japan; 8 Sportology Center, Graduate School of Medicine, Juntendo University, 2-1-1 Hongo, Bunkyo-ku, Tokyo 113-8421, Japan; National Cancer Institute, National Institutes of Health, United States of America

## Abstract

**Aims:**

The *DUSP9* locus on chromosome X was identified as a susceptibility locus for type 2 diabetes in a meta-analysis of European genome-wide association studies (GWAS), and GWAS in South Asian populations identified 6 additional single nucleotide polymorphism (SNP) loci for type 2 diabetes. However, the association of these loci with type 2 diabetes have not been examined in the Japanese. We performed a replication study to investigate the association of these 7 susceptibility loci with type 2 diabetes in the Japanese population.

**Methods:**

We genotyped 11,319 Japanese participants (8,318 with type 2 diabetes and 3,001 controls) for each of the 7 SNPs–rs5945326 near *DUSP9*, rs3923113 near *GRB14*, rs16861329 in *ST6GAL1*, rs1802295 in *VPS26A*, rs7178572 in *HMG20A*, rs2028299 near *AP3S2*, and rs4812829 in *HNF4A*–and examined the association of each of these 7 SNPs with type 2 diabetes by using logistic regression analysis.

**Results:**

All SNPs had the same direction of effect (odds ratio [OR]>1.0) as in the original reports. One SNP, rs5945326 near DUSP9, was significantly associated with type 2 diabetes at a genome-wide significance level (p = 2.21×10^−8^; OR 1.39, 95% confidence interval [CI]: 1.24−1.56). The 6 SNPs derived from South Asian GWAS were not significantly associated with type 2 diabetes in the Japanese population by themselves (*p*≥0.007). However, a genetic risk score constructed from 6 South Asian GWAS derived SNPs was significantly associated with Japanese type 2 diabetes (*p* = 8.69×10^−4^, OR  = 1.06. 95% CI; 1.03−1.10).

**Conclusions/interpretation:**

These results indicate that the *DUSP9* locus is a common susceptibility locus for type 2 diabetes across different ethnicities, and 6 loci identified in South Asian GWAS also have significant effect on susceptibility to Japanese type 2 diabetes.

## Introduction

Diabetes mellitus affects more than 300 million individuals worldwide, and its prevalence is progressively increasing, particularly in East Asian and Western Pacific regions [1]. Type 2 diabetes is the most common form of the disease, and the combination of insulin resistance in peripheral tissues and impaired insulin secretion from pancreatic β cells is believed to contribute to the development and progression of type 2 diabetes. Although the precise mechanisms responsible for the onset of the disease are still unknown, multiple genetic factors may cooperate with environmental factors to confer susceptibility to type 2 diabetes [2].

Nearly 40 susceptibility loci have been identified and confirmed for type 2 diabetes in populations of European descent [3]–[15]. Among them, several loci, including *TCF7L2*, *CDKAL1*, *HHEX*, *SLC30A8*, *KCNJ11, CDKN2A*/*B*, *IGF2BP2*, *GCKR*, *IRS1*, and *CDC123*/*CAMK1D*, have been shown to be associated with type 2 diabetes in different ethnic groups [4],[16]–[21]. Japanese genome-wide association studies (GWAS) have identified the loci *KCNQ1*, *UBE2E2*, *C2CD4A-C2CD4B,* and *ANK1* to be associated with type 2 diabetes [22]–[25]. All these loci, except *UBE2E2*, were also shown to be associated with type 2 diabetes in European populations [22]–[25]. Therefore, it is important to examine multiple ethnic populations through GWAS for the identification of ethnicity-specific loci as well as common susceptibility loci. Recent GWAS in East Asian populations and South Asian populations have identified 14 additional susceptibility loci for type 2 diabetes–8 in the East Asian study [26] and 6 in the South Asian study [27]. Thus, the number of susceptibility loci for type 2 diabetes have increased through GWAS to nearly 60, contributing to a better understanding of type 2 diabetes heritability; however, the currently available genetic information explains only ∼10% of this heritability [15],[28].

An expanded meta-analysis of the existing European GWAS data (DIAGRAM+: Diabetes Genetics Replication and Meta-analysis+) has identified 12 loci, including 11 autosomal loci and the *DUSP9* locus on chromosome X, as a strong susceptibility locus for type 2 diabetes [15]. *DUSP9* encodes a member of the family of mitogen-activated protein kinase phosphatase 4, MKP4, which was also shown to play pivotal roles in regulating insulin actions [29],[30],[31]. Although the *DUSP9* locus is considered an important locus for conferring susceptibility to insulin resistance, the contribution of this locus to type 2 diabetes susceptibility in the Japanese population has not yet been evaluated.

In this study, we aim to evaluate the association of 7 loci, which have not been evaluated in Japanese populations, with susceptibility to type 2 diabetes in a Japanese population: 1 single nucleotide polymorphism (SNP) in *DUSP9* on chromosome X and 6 South Asian GWAS-derived loci–*GRB14*, *ST6GAL1*, *VPS26A*, *HMG20A*, *AP3S2*, and *HNF4A*.

## Materials and Methods

### Participants and DNA Preparation

We enrolled 8,318 type 2 diabetes patients who regularly visited the outpatient clinics of the Shiga University of Medical Science, Kawasaki Medical School, St. Marianna University, Juntendo University, and the University of Toyama or who were registered in BioBank Japan [24]. Diabetes mellitus was diagnosed by the World Health Organization (WHO) criteria [32], and type 2 diabetes was defined by a gradual adult onset. Patients who tested positive for antibodies to glutamic acid decarboxylase or who were diagnosed with mitochondrial disease or maturity-onset diabetes of the young were excluded from the present study. We also recruited 3,001 controls who underwent annual health check-ups at Keio University, St. Marianna University, or Toyama University Hospital or from the general Japanese population registered in the Japanese SNP database [24]. Obesity was defined as individuals with BMI ≥25 according to the criteria of Japan society for the study of obesity [33].

### Ethics Statements

All participants agreed to the protocol of this study and provided written informed consent. DNA samples were obtained from the peripheral blood of each participant, and the study protocol was approved by the ethics committees of RIKEN Yokohama Institutes and each of the participating institutes, i.e. Shiga University of Medical Science, Kawasaki Medical School, St. Marianna University, Juntendo University, the University of Toyama, and Keio University.

### SNP Genotyping

We selected 6 autosomal SNPs identified by GWAS in individuals of South Asian ancestry, which were reported by Kooner et al [27]. These included rs3923113 near the growth factor receptor-bound protein 14 gene (*GRB14*), rs16861329 in the ST6 beta-galactosamide alpha-2,6-sialyltranferase 1 gene (*ST6GAL1*), rs1802295 in the vacuolar protein-sorting 26 homolog A gene (*VPS26A*), rs7178572 in the high-mobility group 20A gene (*HMG20A*), rs2028299 near the adaptor-related protein complex 3 sigma 2 subunit gene (*AP3S2*), and rs4812829 in the hepatocyte nuclear factor 4 alpha gene (*HNF4A*). We also examined 1 X-chromosomal SNP, rs5945326, near the dual-specificity phosphatase 9 gene (*DUSP9*), which was identified in a European GWAS reported by Voight et al [15]. The 11 autosomal SNPs also reported by Voight et al. [15] have been already evaluated in the present samples [20], and not examined in the present study.

Genotyping was performed using the multiplex-polymerase chain reaction (PCR) invader assay [34]. Genotyping success rates for all SNPs were over 98% (98.3% for rs3923113, 98.8% for rs16861329, 98.8% for rs1802295, 99.1% for rs7178572, 99.1% for rs2028299, 98.9% for rs4812829, 98.9% for rs5945326), and concordance rates for all SNPs in 95 randomly selected duplicated samples are 100%. Since our samples have been collected in 6 distinct study groups, we compared the risk allele frequencies of the 7 SNPs among the 6 groups. The results indicated, risk allele frequencies of each SNPs were not different among the 6 groups, except for rs5945326, of which risk allele frequency in group 6 control was lower than those in other controls (Table S1).

### Statistical Analysis

We performed Hardy-Weinberg equilibrium (HWE) tests according to the method described by Nielsen et al [35]. Differences in the genotype distribution of each SNP between cases and controls were evaluated by logistic regression analysis with or without adjusting for age, sex, and body mass index (BMI). The association of each SNP with quantitative traits, BMI, fasting plasma glucose (FPG), the homeostasis model assessment of β-cell function (HOMA-β), and the HOMA of insulin resistance (HOMA-IR) [36],[37] was evaluated by multiple linear regression analysis. Because values of these traits in the present Japanese population showed a skewed distribution, we used log-transformed values for the analyses. Genotypes of autosomal SNPs were scored using an additive model (0, 1, and 2 for the homozygous of non-effect allele, heterozygous, and homozygous of effect allele, respectively). For evaluating X-chromosome SNP, men were coded as homozygous, and all men and homozygous women were included in the association study (0 for the homozygous of non-effect allele and 1 for the homozygous of effect allele, assuming X-chromosome inactivation in females.).

Statistical analyses were performed using StatView software (SAS Institute, Cary, NC, USA). The level of significance was determined by the Bonferroni’s method for correcting multiple testing errors, and a *p* value of <0.007 (0.05 divided by 7) was considered statistically significant.

## Results

### Characteristics of the Study Participants are Shown in Table 1

Genotype distributions of all autosomal SNPs in the present study were in accordance with the Hardy-Weinberg equilibrium proportions (Table 2). The results of the association study for each SNP with type 2 diabetes in the present Japanese population are shown in Table 3. All SNPs had the same direction of effect (odds ratio [OR] >1.0) as in the original reports [15],[27]. Of these, 1 SNP, rs5945326 near *DUSP9*, was significantly associated with type 2 diabetes, and the association reached a genome-wide significance level (*p*<5×10^−8^). A subsequent sex-stratified analysis for rs5945326 showed no remarkable difference in the effect size between men and women (OR in women: 1.45, 95% confidence interval [CI]: 1.12−1.87 OR in men: 1.38, 95%CI: 1.21−1.57 Table S2). One SNP, rs7178572 in *HMG20A*, had a nominal association with type 2 diabetes (*p*<0.05, Table 3); however, this association was not significant after Bonferroni’s correction. The remaining 5 SNPs were not associated with type 2 diabetes in the Japanese population (*p*≥0.05). We did not observe a significant association of 6 autosomal SNPs with type 2 diabetes in a dominant or recessive association model (Table S3, Table S4). However, a genetic risk score (GRS) constructed from the 6 SNPs was significantly associated with type 2 diabetes in the present Japanese population (Table 3).

**Table 1:** Characteristics of participants.

	Sample size (case/control)	Type 2 diabetes	Controls	*p* value
n		8,318	3,001	
Sex (M:F)		5,098:3,220	1,670:1,331	<0.001^b^
Age (year)^a^	8,281/2,995	63.6±11.1	50.5±16.3	<0.001^c^
BMI (kg/m^2^)^a^	7,948/2,954	24.2±4.0	22.8±3.2	<0.001^c^
HbA1c (%)^a^	5,199/1,388	7.9±2.0	5.4±0.3	<0.001^c^
PG (mmol/L)^a^	2,376/1,375	8.5±3.1	5.3±0.6	<0.001^c^
TC (mmol/L)^a^	2,084/1,553	5.2±0.9	5.3±0.9	<0.001^c^
TG (mmol/L)^a^	2,064/1,616	1.4±0.4	1.2±0.8	<0.001^c^
HDL-C (mmol/L)^a^	2,304/1,460	1.4±0.4	1.6±0.4	<0.001^c^
SBP (mmHg)^a^	3,938/2,469	134±17	128±18	<0.001^c^
Duration of diabetes^a^	6,704/−	11.8±9.2	–	

a Data are mean ± SD.

b Chi-square test.

c Student’s unpaired t-test.

BMI: body mass index, HbA1c: Glycated hemoglobin, PG: plasma glucose, TC: total cholesterol, TG: triglyceride, HDL-C: high density lipoprotein cholesterol, SBP: systolic blood pressure.

**Table 2:** Genotype distributions for 7 SNPs in case and control groups.

SNP	Gene	Allele1/Allele2	Allele 11/12/22		P for HWE test	
			Type 2 diabetes	Controls	Type 2 diabetes	Controls
rs3923113	*GRB14*	A/C	6613/1448/86	2368/582/30	0.4987	0.3830
rs16861329	*ST6GAL1*	G/A	5399/2518/309	1877/977/106	0.4697	0.1248
rs1802295	*VPS26A*	G/A	6582/1538/97	2398/544/28	0.5025	0.6417
rs7178572	*HMG20A*	A/G	2785/4003/1449	1070/1438/472	0.8714	0.7600
rs2028299	*AP3S2*	A/C	4892/2910/428	1798/1049/138	0.8599	0.3381
rs4812829	*HNF4A*	G/A	2377/4151/1712	912/1445/603	0.1996	0.4831
rs5945326	*DUSP9*	A/G	5153/0/1704^a^	1678/0/740 a	–	–

a Males are coded as homozygous, and homozygous women and all men are included in the analysis.

**Table 3:** Association of 7 SNPs with type 2 diabetes in the Japanese population.

SNP	Gene	Risk Allele^a^	RAF^b^(case/control)	Unadjusted		Adjusted^c^	
				*p* value	OR (95%CI)	*p* value	OR (95%CI)
rs3923113	*GRB14*	A	0.901/0.892	0.0700	1.09 (0.99–1.20)	0.0779	1.10 (0.99–1.23)
rs16861329	*ST6GAL1*	G	0.809/0.799	0.0872	1.07 (0.99–1.15)	0.1526	1.06 (0.98–1.16)
rs1802295	*VPS26A*	A	0.105/0.101	0.3444	1.05 (0.95–1.16)	0.8579	1.01 (0.90–1.13)
rs7178572	*HMG20A*	G	0.419/0.400	0.0098	1.08 (1.02–1.15)	0.0248	1.08 (1.01–1.16)
rs2028299	*AP3S2*	C	0.229/0.222	0.2774	1.04 (0.97–1.12)	0.3352	1.04 (0.96–1.13)
rs4812829	*HNF4A*	A	0.460/0.448	0.1154	1.05 (0.99–1.11)	0.1386	1.05 (0.98–1.13)
GRS^d^				1.05×10^−4^	1.06 (1.03−1.10)	8.69×10^−4^	1.06 (1.03−1.10)
rs5945326	*DUSP9*	A	0.751/0.694	3.55×10^−8^	1.33 (1.20–1.48)	2.21×10^−8^	1.39 (1.24–1.56)

Results of logistic regression analysis are shown.

a risk allele reported in the previous reports.

b risk allele frequency.

c adjusted for age,sex and log-transformed BMI.

d GRS was calculated according to the number of risk alleles by counting the 6 South Asian GWAS derived SNPs.

We next examined the associations between these SNPs and quantitative traits related to glucose metabolism, such as HOMA-IR, HOMA-β, and FPG, by using the control samples. The A-allele of rs5945326 (near the *DUSP9*) had a modest effect on increased HOMA-IR and HOMA-β values (Table 4, Table S5). None of the SNPs showed significant association with these glycemic traits in the present Japanese population, although the sample size might not be sufficiently large enough (Table S6). We further searched MAGIC (the Meta-Analyses of Glucose and Insulin-related traits Consortium) data for 6 South Asian GWAS derived SNPs. Although rs3923113 near *GRB14* has modest effect on reduced insulin sensitivity (HOMA-IR, β = 0.011, se = 0.0042, p = 0.01275), we did not find significant association between the 6 SNPs and glycemic traits (Table S7).

**Table 4:** Association of 7 SNPs with quantitative traits related to glucose metabolism in controls.

SNP	Gene	Risk Allele^a^	HOMA-IR^b^ (n = 900)		HOMA-ß^b^(n = 900)		FPG^b^(n = 1,332)	
			Effect (SE)	*p* value	Effect (SE)	*p* value	Effect (SE)	*p* value
rs3923113	*GRB14*	A	−0.021 (0.037)	0.5613	−0.002 (0.039)	0.9548	−0.002 (0.006)	0.7217
rs16861329	*ST6GAL1*	G	−0.015 (0.028)	0.5955	−0.026 (0.03)	0.3886	−0.003 (0.004)	0.4933
rs1802295	*VPS26A*	A	0.044 (0.038)	0.2436	−0.04 (0.04)	0.3164	0.009(0.006)	0.1231
rs7178572	*HMG20A*	G	0.031 (0.023)	0.1762	0.026 (0.025)	0. 2982	0.001 (0.004)	0.7469
rs2028299	*AP3S2*	C	−0.035 (0.027)	0.1937	−0.003 (0.028)	0. 9045	−0.00005 (0.004)	0.9897
rs4812829	*HNF4A*	A	−0.004 (0.022)	0.8527	0.008 (0.024)	0. 7495	0.003 (0.004)	0.4398
GRS^c^			−0.001 (0.012)	0.9242	0.0002 (0.012)	0.9891	0.001 (0.002)	0.6041
rs5945326	*DUSP9*	A	0.066 (0.037)	0.0769	0.073 (0.039)	0.063	−0.006 (0.006)	0.2783
		BMI<25	0.099 (0.042)	0.0198	0.105 (0.046)	0.0214	−0.006 (0.007)	0.3515
		BMI≥25	−0.035 (0.076)	0.6423	−0.016 (0.074)	0.8293	−0.008 (0.012)	0.5261

Results of linear regression analysis with adjusting age, sex and log-transformed BMI are presented.

a risk allele for type 2 diabetes reported in the previous reports.

b values are log-transformed for the analysis.

Using the statistics were stratified at BMI 25.

c GRS was calculated according to the number of risk alleles by counting the above 6 SNPs (n = 868 for HOMA-IR and HOMA-ß, n = 1,236 for FPG).

We also examined the association between these SNPs and BMI (Table 5, Table S8). The results indicated that rs2028299 near *AP3S2* was significantly associated with BMI only when both patients and controls were used for the analysis, with adjustment for age, sex, and disease state of type 2 diabetes (rs2028299, β = −0.007, SE = 0.003, *p* = 0.0032; Table 5).

**Table 5:** Association of 7 SNPs with BMI in the Japanese population.

SNP	Gene	Risk Allele^a^	All participants (cases & controls)^b^(n = 10,902)		Cases^c^ (n = 7,948)		Controls^c^ (n = 2,954)	
			Effect (SE)	*p* value	Effect (SE)	*p* value	Effect (SE)	*p* value
rs3923113	*GRB14*	A	−0.003 (0.003)	0.4097	−0.005 (0.004)	0.2664	0.002 (0.005)	0.7326
rs16861329	*ST6GAL1*	G	0.003 (0.003)	0.2607	0.003 (0.003)	0.2876	0.004 (0.004)	0.3351
rs1802295	*VPS26A*	A	0.003 (0.003)	0.3181	0.001 (0.004)	0.7320	0.004(0.006)	0.4416
rs7178572	*HMG20A*	G	−0.002 (0.002)	0.3443	−0.004 (0.003)	0.1590	0.003 (0.003)	0.4463
rs2028299	*AP3S2*	C	−0.007 (0.003)	0.0032	−0.008 (0.003)	0.0085	−0.003 (0.004)	0.3929
rs4812829	*HNF4A*	A	0.001 (0.002)	0.7559	0.001 (0.002)	0.5875	−0.001 (0.003)	0.7348
rs5945326	*DUSP9*	A	−0.005 (0.004)	0.1640	−0.006 (0.004)	0.1703	−0.003 (0.006)	0.6100

Results of linear regression analysis are presented. Log-transformed BMI was used for the analysis.

a risk allele reported in the previous reports.

b adjusted for age, sex and disease state of type 2 diabetes (control = 0, case = 1).

c adjusted for age and sex.

## Discussion

In this study, we examined the association of 7 SNPs, previously identified by European or South Asian GWAS, with susceptibility to type 2 diabetes in a Japanese population and showed that rs5945326 near *DUSP9*, an X-chromosome SNP, was significantly associated with type 2 diabetes in the Japanese population.

To date, nearly 60 susceptibility loci have been identified for type 2 diabetes [3]–[15],[22]–[27],[38]. Of these, several loci have been shown to be associated with type 2 diabetes in different ethnic groups, whereas the remaining loci have not shown significant effects in ethnic populations other than those in the original reports. Although the power of these studies may be insufficient in some cases, genetic heterogeneity among different ethnicities may exist for some particular loci.

Among the examined SNP loci in the present study, we found that rs5945326 near *DUSP9* on the X chromosome was strongly associated with type 2 diabetes in the Japanese population. The association of rs5945326 attained a genome-wide significance (*p*<5.0×10^−8^) in the present sample, and the risk allele (A) was in accordance with that in the original report [15]. Furthermore, the association of rs5945326 with type 2 diabetes was also observed in Pakistani [39] or in Han Chinese population [40], indicating that this locus is a common susceptibility locus for type 2 diabetes across multiple ethnic groups. Furthermore, the effect size (OR) of rs5945326-A in the present Japanese sample (OR = 1.39) was larger than that in other Japanese type 2 diabetes loci, except for *KCNQ1* and *TCF7L2* [17],[18],[22],[23]. This result suggests that the *DUSP9* locus is one of the most important susceptibility loci for type 2 diabetes in the Japanese population. Although the risk allele frequency of rs5945326 is lower in one control group than those in other control groups, the association of rs5945326 was still statistically significant even after excluding the control (group 6 control, Table S1) (*p* = 2.95×10^−4^, OR = 1.30, 95% CI; 1.13–1.49); therefore, we thought that the present positive finding was not affected by a sample heterogeneity.

*DUSP9* encodes a dual-specificity phosphatase 9 (MKP4). MKP4 is expressed in insulin-responsive tissues, including adipose, muscle, and liver tissues. Emanuelli et al. showed that MKP4 has protective effect against the development of insulin resistance because of its ability to inactivate extracellular signal-regulated kinase (ERK) or c-Jun N-terminal kinase (JNK), which are considered to be the mediators of stress-induced insulin resistance [31]. In this report, Emanuelli et al. also showed that overexpression of *Mkp4* in the liver by injecting adenovirus vectors encoding *Mkp4* ameliorated insulin resistance in ob/ob mice, suggesting that Mkp4/Dusp9 has favourable effects on glucose metabolism by increasing insulin sensitivity. In contrast, other research groups have shown that MKP4 decreases insulin-stimulated glucose uptake in cultured adipocytes [29],[30]. Therefore, the effects of MKP4/DUSP9 on the metabolic action of insulin may differ between different tissues or under different conditions. Regardless, MKP4 can be considered an important regulator of insulin sensitivity. In the present study, we observed that a risk allele of rs5945326 near *DUSP9* tended to increase HOMA-IR (β = 0.066, *p* = 0.0769; Table 4), and subsequent BMI-stratified analysis showed that the effects of the rs5945326-A allele could be observed only in the non-obese group (BMI<25, β = 0.099, *p* = 0.0198; Table 4, Table S9) but not in obese group (BMI≥25, β = -0.035, *p* = 0.6423); the effect of rs5945326-A on reducing insulin sensitivity might be masked by obesity-induced insulin resistance in the obese group. Although the association of rs5945326 with HOMA-IR was still not statistically significant after correction for multiple testing errors, the rs5945326-A allele may confer susceptibility to type 2 diabetes by reducing insulin sensitivity.

Regarding the remaining 6 loci derived from a South Asian GWAS, rs7178572 in the intron of *HMG20A* was nominally associated with type 2 diabetes; however, no SNPs showed a significant association with type 2 diabetes in the present Japanese population after correction for multiple testing errors. Because all SNP loci had the same direction of effect as in the original reports (OR>1.0) and the estimated powers in the present study for these unreplicated SNP loci were between 28% and 76% (Table S10), insufficient power of the present study may be the principal explanation for the differences between the present study and the original study in South Asian populations. Then we further examined a combinational effect of these 6 loci using GRS constructed from the 6 South Asian GWAS derived SNPs, and found that the GRS was significantly associated with Japanese type 2 diabetes (Table 3), suggesting these loci also have significant effect on susceptibility to Japanese type 2 diabetes.

Because control subjects in the present study were younger than type 2 diabetes patients, the control group might include several individuals who would develop the disease, and the possibility of type 2 error may be increased, although results were not affected by adjusting age. Then we evaluated the association of the 7 SNPs with type 2 diabetes using older control individuals (age≥50 or ≥60). The results were almost the same as original findings, although effect sizes for rs3923113 and rs16861329 on the disease susceptibility were likely increased when we selected older control individuals for the association study (Table S11). In addition, there were considerable differences in the risk allele frequencies for these loci between the Japanese and South Asian populations (Table S12).

In a subsequent study to determine the association of all 7 SNPs with BMI, we observed a significant association of rs2028299 near *AP3S2* with BMI when the analysis was performed using all participants, i.e. both type 2 diabetes patients and controls. *AP3S2* encodes a clathrin-associated adaptor complex. The expression of *AP3S2* has been shown to be ubiquitous, and no report has yet emerged to suggest a functional role of this gene product in the pathogenesis of obesity or type 2 diabetes. There are several nearby genes (other than *AP3S2*) around rs2028299, including *PLIN1* that lies 200 kb from rs2028299 and encodes perilipin-1, a hormonally regulated phosphoprotein that coats fat droplets [41]. Because perilipin has been shown to play pivotal roles in adipocyte lipid metabolism and has been shown to have an association with obesity in humans and experimental animal models [42]–[45], the present signal may reflect the effects of nearby genes, including *PLIN1*. However, the association of rs2028299 with BMI in the present study was modest, and a larger association study is required to clarify the association of this locus with BMI.

In conclusion, we observed a significant association of rs5945326 with type 2 diabetes in a Japanese population. The results indicate that rs5945326 is a common type 2 diabetes susceptibility locus across different ethnic groups. Although it is suggested that the 6 South Asian GWAS-derived loci have significant effect on conferring susceptibility to type 2 diabetes in the Japanese, studies with larger cohorts from the Japanese population are required for evaluating the association of the 6 loci with type 2 diabetes. Moreover, further functional studies are necessary to elucidate the biological mechanisms of each locus for conferring susceptibility to the disease.

## Supporting Information

Table S1 **Comparison of risk allele frequencies among individual areas for sample collection.** ^a^collection 1 (case [Shiga University of Medical Science, Kawasaki Medical School], control [Keio University]), collection 2 (St. Marianna University), collection 3 (Toyama University), collection 4 (Juntendo University), collection 5 (BioBank Japan 1), collection 6 (BioBank Japan 2). ^b^Chi square test.
(DOC)

Table S2 **Sex stratified analysis for the association of rs5945326 near** ***DUSP9*** **with type 2 diabetes.** ^a^adjusted for age and log-transformed BMI.
(DOC)

Table S3 **Association of 6 autosomal SNPs with type 2 diabetes in the Japanese population by using a dominant association model.** Results of logistic regression analysis are shown. ^a^risk allele reported in the previous reports. ^b^adjusted for age, sex and log-transformed BMI.
(DOC)

Table S4 **Association of 6 autosomal SNPs with type 2 diabetes in the Japanese population by using a recessive association model.** Results of logistic regression analysis are shown. ^a^risk allele reported in the previous reports. ^b^adjusted for age,sex and log-transformed BMI.
(DOC)

Table S5 **Sex stratified analysis for the association of rs5945326 near** ***DUSP9*** **with quantitative traits related to glucose metabolism in controls.** Results of linear regression analysis with adjusting age and log-transformed BMI are presented. ^a^values are log-transformed for the analyses.
(DOC)

Table S6 **Estimation of statistical power for the present study to detect associations of 7 SNPs with quantitative metabolic traits.** Power calculations were carried out using the Quanto software package (Version 1.2.4, http://hydra.usc.edu/gxe/). ^a^values are log-transformed for the analysis.
(DOCX)

Table S7 **Association of 6 SNPs with quantitative traits related to glucose metabolism in European populations.** Data on glycaemic traits have been contributed by MAGIC (the Meta-Analyses of Glucose and Insulin-related traits Consortium) investigators and have been downloaded from www.magicinvestigators.org (Dupuis J et al, New genetic loci implicated in fasting glucose homeostasis and their impact on type 2 diabetes risk. Nat Genet. 2010;42:105–116). ^a^risk allele for type 2 diabetes reported in the previous reports. ^b^values are log-transformed for the analysis.
(DOCX)

Table S8 **Sex stratified analysis for the association of rs5945326 near** ***DUSP9*** **with BMI in the Japanese population.** Results of linear regression analysis are presented. Log-transformed BMI was used for the analysis. ^a^adjusted for age and disease state of type 2 diabetes (control = 0, case = 1). ^b^adjusted for age.
(DOC)

Table S9 **Association of 7 SNPs with quantitative traits related to glucose metabolism in obese controls (BMI≥25) or in non-obese controls (BMI<25).** Results of linear regression analysis with adjusting age,sex and log-transformed BMI are presented. ^a^values are log-transformed for the analysis.
(DOC)

Table S10 **Power estimation for each SNP locus in the present study.** Power estimation was performed using CaTS power calculator, CaTS: http://www.sph.umich.edu/csg/abecasis/CaTS/). The prevalence of type 2 diabetes is assumed to be 10%, α  = 0.05. ^a^risk allele for type 2 diabetes reported in the previous reports.
(DOC)

Table S11 **Association study of 7 SNPs with type 2 diabetes using older control (age ≥ 50, n = 1,640, age ≥ 60 n = 930) and all cases (n = 8,318).** Results of logistic regression analysis are shown. ^a^risk allele reported in the previous reports.
(DOC)

Table S12 **Comparison of risk allele frequencies or effect sizes for 6 loci between the Japanese and South Asian (SA) populations.** ^a^Data from the previous report (Kooner et al. Nat Genet 43:984–989, 2011).
(DOC)
