# Supplementary material for: A Single Nucleotide Polymorphism within DUSP9 Is Associated with Susceptibility to Type 2 Diabetes in a Japanese Population
Source: PLoS One. 2012 Sep 27;7(9):e46263. doi: 10.1371/journal.pone.0046263 (PMC3459833; doi:10.1371/journal.pone.0046263)
Supplement: Table S1 — Comparison of risk allele frequencies among individual areas for sample collection. acollection 1 (case [Shiga University of Medical Science, Kawasaki Medical School], control [Keio University]), collection 2 (St. Marianna University), collection 3 (Toyama University), collection 4 (Juntendo University), collection 5 (BioBank Japan 1), collection 6 (BioBank Japan 2). bChi square test. (DOC) [file pone.0046263.s001.doc]

**Table S1** Comparison of risk allele frequencies among individual areas for sample collection

|  |  | Risk allele frequencies | | | | | |  |
| --- | --- | --- | --- | --- | --- | --- | --- | --- |
| SNP | collection^a^ | 1 | 2 | 3 | 4 | 5 | 6 | *p*^b^ |
| rs3923113 | case | 0.895 | 0.899 | 0.897 | 0.903 | 0.901 | 0.903 | 0.4333 |
|  | control | 0.908 | 0.892 | 0.886 |  |  | 0.886 | 0.2715 |
| rs16861329 | case | 0.815 | 0.815 | 0.827 | 0.816 | 0.814 | 0.797 | 0.0770 |
|  | control | 0.799 | 0.800 | 0.787 |  |  | 0.805 | 0.4440 |
| rs1802295 | case | 0.107 | 0.100 | 0.102 | 0.110 | 0.118 | 0.100 | 0.2117 |
|  | control | 0.103 | 0.090 | 0.106 |  |  | 0.102 | 0.6684 |
| rs7178572 | case | 0.430 | 0.438 | 0.420 | 0.412 | 0.434 | 0.409 | 0.3426 |
|  | control | 0.408 | 0.376 | 0.400 |  |  | 0.408 | 0.4756 |
| rs2028299 | case | 0.232 | 0.243 | 0.213 | 0.225 | 0.222 | 0.233 | 0.8186 |
|  | control | 0.228 | 0.211 | 0.228 |  |  | 0.220 | 0.2202 |
| rs4812829 | case | 0.454 | 0.447 | 0.461 | 0.468 | 0.438 | 0.465 | 0.6398 |
|  | control | 0.461 | 0.445 | 0.448 |  |  | 0.440 | 0.5110 |
| rs5945326 | case | 0.747 | 0.725 | 0.725 | 0.755 | 0.777 | 0.754 | 0.2717 |
|  | control | 0.702 | 0.725 | 0.711 |  | 0.659 |  | 0.0492 |

^a^collection 1 (case [Shiga University of Medical Science, Kawasaki Medical School], control [Keio University]), collection 2 (St. Marianna University), collection 3 (Toyama University), collection 4 (Juntendo University), collection 5 (BioBank Japan 1), collection 6 (BioBank Japan 2)

^b^Chi square test
