# Supplementary material for: A Single Nucleotide Polymorphism within DUSP9 Is Associated with Susceptibility to Type 2 Diabetes in a Japanese Population
Source: PLoS One. 2012 Sep 27;7(9):e46263. doi: 10.1371/journal.pone.0046263 (PMC3459833; doi:10.1371/journal.pone.0046263)
Supplement: Table S2 — Sex stratified analysis for the association of rs5945326 near DUSP9 with type 2 diabetes. aadjusted for age and log-transformed BMI. (DOC) [file pone.0046263.s002.doc]

**Table S2** Sex stratified analysis for the association of rs5945326 near *DUSP9* with type 2 diabetes

|  |  | Unadjusted | | Adjusted^a^ | |
| --- | --- | --- | --- | --- | --- |
|  | RAF (case/control) | *p* value | OR(95%CI) | *p* value | OR (95%CI) |
| Women | 0.834/0.772 | 0.0002 | 1.48 (1.21–1.83) | 0.0048 | 1.45 (1.12–1.87) |
| Men | 0.721/0.657 | 7.71×10^-7^ | 1.35 (1.20–1.52) | 1.52×10^-6^ | 1.38 (1.21–1.57) |

^a^adjusted for age and log-transformed BMI
