# Supplementary material for: A Single Nucleotide Polymorphism within DUSP9 Is Associated with Susceptibility to Type 2 Diabetes in a Japanese Population
Source: PLoS One. 2012 Sep 27;7(9):e46263. doi: 10.1371/journal.pone.0046263 (PMC3459833; doi:10.1371/journal.pone.0046263)
Supplement: Table S3 — Association of 6 autosomal SNPs with type 2 diabetes in the Japanese population by using a dominant association model. Results of logistic regression analysis are shown. arisk allele reported in the previous reports. badjusted for age, sex and log-transformed BMI. (DOC) [file pone.0046263.s003.doc]

**Table S3** Association of 6 autosomal SNPs with type 2 diabetes in the Japanese population by using a dominant association model

| SNP | Gene | Risk Allele^a^ | Unadjusted | | Adjusted^b^ | |
| --- | --- | --- | --- | --- | --- | --- |
|  |  |  | *p* value | OR(95%CI) | *p* value | OR (95%CI) |
| rs3923113 | *GRB14* | A | 0.8221 | 0.95 (0.63–1.45) | 0.6483 | 1.12 (0.70–1.78) |
| rs16861329 | *ST6GAL1* | G | 0.6652 | 0.95 (0.76–1.19) | 0.5522 | 0.93 (0.72–1.20) |
| rs1802295 | *VPS26A* | A | 0.4356 | 1.04 (0.94–1.16) | 0.9236 | 1.01 (0.89–1.13) |
| rs7178572 | *HMG20A* | G | 0.0391 | 1.10 (1.00–1.20) | 0.0690 | 1.10 (0.99–1.21) |
| rs2028299 | *AP3S2* | C | 0.4491 | 1.03 (0.95–1.13) | 0.3700 | 1.05 (0.95–1.15) |
| rs4812829 | *HNF4A* | A | 0.0442 | 1.10 (1.00–1.20) | 0.0733 | 1.10 (0.99–1.22) |

Results of logistic regression analysis are shown

^a^risk allele reported in the previous reports

^b^adjusted for age, sex and log-transformed BMI.
