# Supplementary material for: A Single Nucleotide Polymorphism within DUSP9 Is Associated with Susceptibility to Type 2 Diabetes in a Japanese Population
Source: PLoS One. 2012 Sep 27;7(9):e46263. doi: 10.1371/journal.pone.0046263 (PMC3459833; doi:10.1371/journal.pone.0046263)
Supplement: Table S4 — Association of 6 autosomal SNPs with type 2 diabetes in the Japanese population by using a recessive association model. Results of logistic regression analysis are shown. arisk allele reported in the previous reports. badjusted for age,sex and log-transformed BMI. (DOC) [file pone.0046263.s004.doc]

**Table S4** Association of 6 autosomal SNPs with type 2 diabetes in the Japanese population by using a recessive association model

| SNP | Gene | Risk Allele^a^ | Unadjusted | | Adjusted^b^ | |
| --- | --- | --- | --- | --- | --- | --- |
|  |  |  | *p* value | OR(95%CI) | *p* value | OR (95%CI) |
| rs3923113 | *GRB14* | A | 0.0432 | 1.11 (1.00–1.24) | 0.0735 | 1.12 (0.99–1.26) |
| rs16861329 | *ST6GAL1* | G | 0.0298 | 1.10 (1.01–1.20) | 0.0564 | 1.10 (0.997–1.22) |
| rs1802295 | *VPS26A* | A | 0.2918 | 1.26 (0.82–1.92) | 0.7097 | 1.09 (0.69–1.73) |
| rs7178572 | *HMG20A* | G | 0.0296 | 1.13 (1.01–1.27) | 0.0638 | 1.13 (0.99–1.29) |
| rs2028299 | *AP3S2* | C | 0.2173 | 1.13 (0.93–1.38) | 0.5554 | 1.07 (0.86–1.33) |
| rs4812829 | *HNF4A* | A | 0.6406 | 1.03 (0.92–1.14) | 0.5796 | 1.03 (0.92–1.16) |

Results of logistic regression analysis are shown

^a^risk allele reported in the previous reports

^b^adjusted for age,sex and log-transformed BMI.
