# Supplementary material for: A Single Nucleotide Polymorphism within DUSP9 Is Associated with Susceptibility to Type 2 Diabetes in a Japanese Population
Source: PLoS One. 2012 Sep 27;7(9):e46263. doi: 10.1371/journal.pone.0046263 (PMC3459833; doi:10.1371/journal.pone.0046263)
Supplement: Table S5 — Sex stratified analysis for the association of rs5945326 near DUSP9 with quantitative traits related to glucose metabolism in controls. Results of linear regression analysis with adjusting age and log-transformed BMI are presented. avalues are log-transformed for the analyses. (DOC) [file pone.0046263.s005.doc]

**Table S5** Sex stratified analysis for the association of rs5945326 near *DUSP9* with quantitative traits related to glucose metabolism in controls

|  | HOMA-IR^a^ | | HOMA-β^a^ | | FPG^a^ | |
| --- | --- | --- | --- | --- | --- | --- |
|  | Effect (SE) | *p* value | Effect (SE) | *p* value | Effect (SE) | *p* value |
| Women | 0.075 (0.076) | 0.3277 | -0.0006 (0.079) | 0.9942 | 0.0010 (0.012) | 0.9377 |
| Men | 0.061 (0.042) | 0.1505 | 0.094 (0.045) | 0.0346 | -0.009 (0.007) | 0.1944 |

Results of linear regression analysis with adjusting age and log-transformed BMI are presented.

^a^values are log-transformed for the analyses.
