# Supplementary material for: A Single Nucleotide Polymorphism within DUSP9 Is Associated with Susceptibility to Type 2 Diabetes in a Japanese Population
Source: PLoS One. 2012 Sep 27;7(9):e46263. doi: 10.1371/journal.pone.0046263 (PMC3459833; doi:10.1371/journal.pone.0046263)
Supplement: Table S6 — Estimation of statistical power for the present study to detect associations of 7 SNPs with quantitative metabolic traits. Power calculations were carried out using the Quanto software package (Version 1.2.4, http://hydra.usc.edu/gxe/). avalues are log-transformed for the analysis. (DOCX) [file pone.0046263.s006.docx]

**Table S6** Estimation of statistical power for the present study to detect associations of 7 SNPs with quantitative metabolic traits

|  | rs3923113 | | | | rs16861329 | | | |
| --- | --- | --- | --- | --- | --- | --- | --- | --- |
| βestimates | HOMA-IR^b^  (n=900) | HOMA-β^b^  (n=900) | FPG^b^  (n=1,332) | BMI  (n=10,902) | HOMA-IR^b^  (n=900) | HOMA-β^b^  (n=900) | FPG^b^  (n=1,332) | BMI  (n=10,902) |
| 0.001 | 0.051 | 0.050 | 0.053 | 0.060 | 0.050 | 0.050 | 0.056 | 0.067 |
| 0.005 | 0.052 | 0.052 | 0.131 | 0.312 | 0.053 | 0.053 | 0.195 | 0.474 |
| 0.01 | 0.057 | 0.056 | 0.378 | 0.835 | 0.062 | 0.061 | 0.593 | 0.966 |
| 0.03 | 0.111 | 0.109 | 0.999 | 1.00 | 0.160 | 0.156 | 1.00 | 1.00 |
| 0.05 | 0.225 | 0.218 | 1.00 | 1.00 | 0.360 | 0.349 | 1.00 | 1.00 |
| 0.1 | 0.672 | 0.656 | 1.00 | 1.00 | 0.894 | 0.883 | 1.00 | 1.00 |
|  | rs1802295 | | | | rs7178572 | | | |
| βestimates | HOMA-IR^a^  (n=900) | HOMA-β^a^  (n=900) | FPG^a^  (n=1,332) | BMI^a^  (n=10,902) | HOMA-IR^b^  (n=900) | HOMA-β^b^  (n=900) | FPG^b^  (n=1,332) | BMI^a^  (n=10,902) |
| 0.001 | 0.050 | 0.050 | 0.053 | 0.059 | 0.050 | 0.050 | 0.058 | 0.075 |
| 0.005 | 0.052 | 0.052 | 0.132 | 0.297 | 0.054 | 0.054 | 0.264 | 0.639 |
| 0.01 | 0.057 | 0.057 | 0.382 | 0.813 | 0.067 | 0.067 | 0.757 | 0.996 |
| 0.03 | 0.112 | 0.110 | 0.999 | 1.00 | 0.213 | 0.207 | 1.00 | 1.00 |
| 0.05 | 0.227 | 0.220 | 1.00 | 1.00 | 0.491 | 0.477 | 1.00 | 1.00 |
| 0.1 | 0.677 | 0.661 | 1.00 | 1.00 | 0.973 | 0.968 | 1.00 | 1.00 |
|  | rs2028299 | | | | rs4812829 | | | |
| βestimates | HOMA-IR^a^  (n=900) | HOMA-β^a^  (n=900) | FPG^a^  (n=1,332) | BMI^a^  (n=10,902) | HOMA-IR^b^  (n=900) | HOMA-β^b^  (n=900) | FPG^b^  (n=1,332) | BMI^a^  (n=10,902) |
| 0.001 | 0.050 | 0.050 | 0.056 | 0.068 | 0.050 | 0.050 | 0.058 | 0.076 |
| 0.005 | 0.053 | 0.053 | 0.206 | 0.502 | 0.054 | 0.054 | 0.271 | 0.652 |
| 0.01 | 0.063 | 0.062 | 0.623 | 0.976 | 0.068 | 0.067 | 0.770 | 0.997 |
| 0.03 | 0.168 | 0.164 | 1.00 | 1.0 | 0.218 | 0.212 | 1.00 | 1.0 |
| 0.05 | 0.381 | 0.369 | 1.00 | 1.0 | 0.502 | 0.488 | 1.00 | 1.0 |
| 0.1 | 0.913 | 0.903 | 1.00 | 1.0 | 0.976 | 0.972 | 1.00 | 1.0 |
|  | rs5945326 | | | |  |  |  |  |
| βestimates | HOMA-IR^a^  (n=900) | HOMA-β^a^  (n=900) | FPG^a^  (n=1,332) | BMI^a^  (n=10,902) |  |  |  |  |
| 0.001 | 0.050 | 0.050 | 0.054 | 0.061 |  |  |  |  |
| 0.005 | 0.052 | 0.052 | 0.140 | 0.338 |  |  |  |  |
| 0.01 | 0.057 | 0.057 | 0.414 | 0.869 |  |  |  |  |
| 0.03 | 0.119 | 0.116 | 0.999 | 1.0 |  |  |  |  |
| 0.05 | 0.246 | 0.239 | 1.00 | 1.0 |  |  |  |  |
| 0.1 | 0.720 | 0.704 | 1.00 | 1.0 |  |  |  |  |

Power calculations were carried out using the Quanto software package (Version 1.2.4, http://hydra.usc.edu/gxe/).

^a^values are log-transformed for the analysis
