# Supplementary material for: A Single Nucleotide Polymorphism within DUSP9 Is Associated with Susceptibility to Type 2 Diabetes in a Japanese Population
Source: PLoS One. 2012 Sep 27;7(9):e46263. doi: 10.1371/journal.pone.0046263 (PMC3459833; doi:10.1371/journal.pone.0046263)
Supplement: Table S7 — Association of 6 SNPs with quantitative traits related to glucose metabolism in European populations. Data on glycaemic traits have been contributed by MAGIC (the Meta-Analyses of Glucose and Insulin-related traits Consortium) investigators and have been downloaded from www.magicinvestigators.org (Dupuis J et al, New genetic loci implicated in fasting glucose homeostasis and their impact on type 2 diabetes risk. Nat Genet. 2010;42:105–116). arisk allele for type 2 diabetes reported in the previous reports. bvalues are log-transformed for the analysis. (DOCX) [file pone.0046263.s007.docx]

**Table S7** Association of 6 SNPs with quantitative traits related to glucose metabolism in European populations

| SNP | Gene | Risk Allele^a^ | HOMA-IR^b^ | | HOMA-β^b^ | | FPG | |
| --- | --- | --- | --- | --- | --- | --- | --- | --- |
|  |  |  | Effect (SE) | *p* value | Effect (SE) | *p* value | Effect (SE) | *p* value |
| rs3923113 | *GRB14* | A | 0.011 (0.004) | 0.013 | 0.006 (0.004) | 0.0936 | -0.004 (0.004) | 0.3666 |
| rs16861329 | *ST6GAL1* | G | 0.003 (0.007) | 0.6978 | 0.002 (0.005) | 0.7482 | 0.0001 (0.0006) | 0.9907 |
| rs1802295 | *VPS26A* | A | -0.0005 (0.004) | 0.9117 | 0.0004 (0.004) | 0.9130 | 0.003 (0.004) | 0.4764 |
| rs7178572 | *HMG20A* | G | -0.001 (0.004) | 0.7407 | -0.004 (0.004) | 0. 2318 | 0.008 (0.004) | 0.0424 |
| rs2028299 | *AP3S2* | C | -0.006 (0.004) | 0.2094 | -0.002 (0.007) | 0. 6409 | -0.002 (0.004) | 0.6131 |
| rs4812829 | *HNF4A* | A | -0.006 (0.006) | 0.2480 | 0.006 (0.004) | 0. 1955 | 0.002 (0.005) | 0.7534 |

Data on glycaemic traits have been contributed by MAGIC (the Meta-Analyses of Glucose and Insulin-related traits Consortium) investigators and have been downloaded from www.magicinvestigators.org (Dupuis J et al, New genetic loci implicated in fasting glucose homeostasis and their impact on type 2 diabetes risk. Nat Genet. 2010;42:105-116)

^a^risk allele for type 2 diabetes reported in the previous reports

^b^values are log-transformed for the analysis
