# Supplementary material for: A Single Nucleotide Polymorphism within DUSP9 Is Associated with Susceptibility to Type 2 Diabetes in a Japanese Population
Source: PLoS One. 2012 Sep 27;7(9):e46263. doi: 10.1371/journal.pone.0046263 (PMC3459833; doi:10.1371/journal.pone.0046263)
Supplement: Table S8 — Sex stratified analysis for the association of rs5945326 near DUSP9 with BMI in the Japanese population. Results of linear regression analysis are presented. Log-transformed BMI was used for the analysis. aadjusted for age and disease state of type 2 diabetes (control = 0, case = 1). badjusted for age. (DOC) [file pone.0046263.s008.doc]

**Table S8** Sex stratified analysis for the association of rs5945326 near *DUSP9* with BMI in the Japanese population

|  | All participants (cases & controls)^a^ | | Cases^b^ | | Controls^b^ | |
| --- | --- | --- | --- | --- | --- | --- |
|  | Effect (SE) | *p* value | Effect (SE) | *p* value | Effect (SE) | *p* value |
| Women | -0.0053 (0.008) | 0.5240 | 0.0023 (0.011) | 0.8373 | -0.013 (0.011) | 0.2542 |
| Men | -0.005 (0.004) | 0.1538 | -0.008 (0.005) | 0.0801 | 0.000473 (0.006) | 0.9420 |

Results of linear regression analysis are presented. Log-transformed BMI was used for the analysis.

^a^adjusted for age and disease state of type 2 diabetes (control=0, case=1)

^b^adjusted for age
