# Supplementary material for: A Single Nucleotide Polymorphism within DUSP9 Is Associated with Susceptibility to Type 2 Diabetes in a Japanese Population
Source: PLoS One. 2012 Sep 27;7(9):e46263. doi: 10.1371/journal.pone.0046263 (PMC3459833; doi:10.1371/journal.pone.0046263)
Supplement: Table S9 — Association of 7 SNPs with quantitative traits related to glucose metabolism in obese controls (BMI≥25) or in non-obese controls (BMI<25). Results of linear regression analysis with adjusting age,sex and log-transformed BMI are presented. avalues are log-transformed for the analysis. (DOC) [file pone.0046263.s009.doc]

**Table S9** Association of 7 SNPs with quantitative traits related to glucose metabolism in obese controls (BMI≥25) or in non-obese controls (BMI<25)

| SNP | Gene |  | HOMA-IR^a^ | | HOMA-β^a^ | | FPG^a^ | |
| --- | --- | --- | --- | --- | --- | --- | --- | --- |
|  |  |  | Effect (SE) | *p* value | Effect (SE) | *p* value | Effect (SE) | *p* value |
| rs3923113 | *GRB14* | BMI<25 | -0.037 (0.041) | 0.3699 | -0.016 (0.044) | 0.7177 | -0.004 (0.007) | 0.5878 |
|  |  | BMI≥25 | 0.063 (0.077) | 0.4139 | 0.084 (0.077) | 0.2787 | 0.004 (0.013) | 0.7743 |
| rs16861329 | *ST6GAL1* | BMI<25 | -0.018 (0.032) | 0.5712 | -0.009 (0.034) | 0.7803 | -0.008 (0.005) | 0.1281 |
|  |  | BMI≥25 | 0.004 (0.059) | 0.9501 | -0.063 (0.058) | 0.2824 | 0.013 (0.010) | 0.1708 |
| rs1802295 | *VPS26A* | BMI<25 | 0.048 (0.042) | 0.2530 | -0.029 (0.044) | 0.5079 | 0.007 (0.007) | 0.2748 |
|  |  | BMI≥25 | 0.040 (0.091) | 0.6623 | -0.061 (0.091) | 0.5055 | 0.017 (0.014) | 0.2314 |
| rs7178572 | *HMG20A* | BMI<25 | 0.027 (0.026) | 0.3047 | 0.009 (0.028) | 0. 7446 | 0.001 (0.004) | 0.7780 |
|  |  | BMI≥25 | 0.009 (0.050) | 0.8610 | 0.028 (0.050) | 0.5812 | 0.005 (0.008) | 0.5526 |
| rs2028299 | *AP3S2* | BMI<25 | -0.024 (0.029) | 0.4156 | -0.002 (0.031) | 0. 9567 | 0.002 (0.005) | 0.6655 |
|  |  | BMI≥25 | -0.082 (0.060) | 0.1732 | -0.028 (0.061) | 0.6495 | -0.009 (0.010) | 0.3359 |
| rs4812829 | *HNF4A* | BMI<25 | -0.014 (0.025) | 0.5753 | 0.003 (0.027) | 0. 9164 | 0.002 (0.004) | 0.5504 |
|  |  | BMI≥25 | 0.019 (0.048) | 0.6918 | 0.010 (0.048) | 0.8335 | 0.005 (0.008) | 0.5576 |
| rs5945326 | *DUSP9* | BMI<25 | 0.099 (0.042) | 0.0198 | 0.105 (0.046) | 0.0214 | -0.006 (0.007) | 0.3515 |
|  |  | BMI≥25 | -0.035 (0.076) | 0.6423 | -0.016 (0.074) | 0.8293 | -0.008 (0.012) | 0.5261 |

Results of linear regression analysis with adjusting age,sex and log-transformed BMI are presented.

^a^values are log-transformed for the analysis
