# Supplementary material for: A Single Nucleotide Polymorphism within DUSP9 Is Associated with Susceptibility to Type 2 Diabetes in a Japanese Population
Source: PLoS One. 2012 Sep 27;7(9):e46263. doi: 10.1371/journal.pone.0046263 (PMC3459833; doi:10.1371/journal.pone.0046263)
Supplement: Table S10 — Power estimation for each SNP locus in the present study. Power estimation was performed using CaTS power calculator, CaTS: http://www.sph.umich.edu/csg/abecasis/CaTS/). The prevalence of type 2 diabetes is assumed to be 10%, α = 0.05. arisk allele for type 2 diabetes reported in the previous reports. (DOC) [file pone.0046263.s010.doc]

**Table S10** Power estimation for each SNP locus in the present study

| SNP | Gene | Risk Allele^a^ | RAF | Reported OR | Power |
| --- | --- | --- | --- | --- | --- |
| rs3923113 | *GRB14* | A | 0.892 | 1.08 | 36% |
| rs16861329 | *ST6GAL1* | G | 0.799 | 1.08 | 47% |
| rs1802295 | *VPS26A* | A | 0.101 | 1.07 | 28% |
| rs7178572 | *HMG20A* | G | 0.400 | 1.08 | 67% |
| rs2028299 | *AP3S2* | C | 0.222 | 1.08 | 56% |
| rs4812829 | *HNF4A* | A | 0.448 | 1.09 | 76% |
| rs5945326 | *DUSP9* | A | 0.694 | 1.27 | 100% |

Power estimation was performed using CaTS power calculator, CaTS: http://www.sph.umich.edu/csg/abecasis/CaTS/)

The prevalence of type 2 diabetes is assumed to be 10%,α = 0.05

^a^risk allele for type 2 diabetes reported in the previous reports
