# Supplementary material for: A Single Nucleotide Polymorphism within DUSP9 Is Associated with Susceptibility to Type 2 Diabetes in a Japanese Population
Source: PLoS One. 2012 Sep 27;7(9):e46263. doi: 10.1371/journal.pone.0046263 (PMC3459833; doi:10.1371/journal.pone.0046263)
Supplement: Table S11 — Association study of 7 SNPs with type 2 diabetes using older control (age ≥ 50, n = 1,640, age ≥ 60 n = 930) and all cases (n = 8,318). Results of logistic regression analysis are shown. arisk allele reported in the previous reports. (DOC) [file pone.0046263.s011.doc]

**Table S11** Association study of 7 SNPs with type 2 diabetes using older control (age ≥ 50, n=1,640, age ≥ 60 n=930) and all cases (n=8,318)

| SNP | Gene | Risk Allele^a^ | Control | Unadjusted | | Adjusted^b^ | |
| --- | --- | --- | --- | --- | --- | --- | --- |
|  |  |  |  | *p* value | OR (95%CI) | *p* value | OR (95%CI) |
| rs3923113 | *GRB14* | A | age ≥ 50 | 0.0491 | 1.13 (1.00–1.27) | 0.763 | 1.12 (0.99–1.26) |
|  |  |  | age ≥ 60 | 0.0388 | 1.17 (1.01–1.36) | 0.0996 | 1.14 (0.98–1.33) |
| rs16861329 | *ST6GAL1* | G | age ≥ 50 | 0.0558 | 1.10 (0.998–1.20) | 0.084 | 1.09 (0.99–1.20) |
|  |  |  | age ≥ 60 | 0.0112 | 1.16 (1.04–1.31) | 0.0127 | 1.17 (1.03–1.31) |
| rs1802295 | *VPS26A* | A | age ≥ 50 | 0.9323 | 0.995 (0.88–1.12) | 0.8006 | 0.98 (0.87–1.11) |
|  |  |  | age ≥ 60 | 0.8433 | 1.02 (0.87–1.19) | 0.9218 | 1.01 (0.86–1.18) |
| rs7178572 | *HMG20A* | G | age ≥ 50 | 0.0526 | 1.08 (0.999–1.17) | 0.0291 | 1.09 (1.01–1.18) |
|  |  |  | age ≥ 60 | 0.0415 | 1.11 (1.00–1.22) | 0.0128 | 1.14 (1.03–1.26) |
| rs2028299 | *AP3S2* | C | age ≥ 50 | 0.5612 | 1.03 (0.94–1.12) | 0.5435 | 1.03 (0.94–1.13) |
|  |  |  | age ≥ 60 | 0.4392 | 1.05 (0.93–1.18) | 0.441 | 1.05 (0.93–1.18) |
| rs4812829 | *HNF4A* | A | age ≥ 50 | 0.1186 | 1.06 (0.99–1.15) | 0.1186 | 1.06 (0.98–1.15) |
|  |  |  | age ≥ 60 | 0.1568 | 1.07 (0.97−1.18) | 0.1358 | 1.08(0.98−1.19) |
| rs5945326 | *DUSP9* | A | age ≥ 50 | 3.64×10^-8^ | 1.43 (1.26–1.62) | 5.51×10^-9^ | 1.47 (1.29–1.67) |
|  |  |  | age ≥ 60 | 7.27×10^-4^ | 1.33 (1.13−1.56) | 7.15×10^-5^ | 1.41 (1.19−1.67) |

Results of logistic regression analysis are shown

^a^risk allele reported in the previous reports

^b^adjusted for age,sex and log-transformed BMI.
