# Supplementary material for: A Single Nucleotide Polymorphism within DUSP9 Is Associated with Susceptibility to Type 2 Diabetes in a Japanese Population
Source: PLoS One. 2012 Sep 27;7(9):e46263. doi: 10.1371/journal.pone.0046263 (PMC3459833; doi:10.1371/journal.pone.0046263)
Supplement: Table S12 — Comparison of risk allele frequencies or effect sizes for 6 loci between the Japanese and South Asian (SA) populations. aData from the previous report (Kooner et al. Nat Genet 43:984–989, 2011). (DOC) [file pone.0046263.s012.doc]

**Table S12** Comparison of risk allele frequencies or effect sizes for 6 loci between the Japanese and South Asian (SA) populations.

| SNP | Gene | Risk Allele |  | RAF | |  | Effect size (odds ratio) | |
| --- | --- | --- | --- | --- | --- | --- | --- | --- |
|  |  |  |  | Japanese | SA ^a^ |  | Japanese | SA ^a^ |
| rs3923113 | *GRB14* | A |  | 0.892 | 0.74 |  | 1.10 | 1.09 |
| rs16861329 | *ST6GAL1* | G |  | 0.799 | 0.75 |  | 1.06 | 1.09 |
| rs1802295 | *VPS26A* | A |  | 0.101 | 0.26 |  | 1.01 | 1.08 |
| rs7178572 | *HMG20A* | G |  | 0.400 | 0.52 |  | 1.08 | 1.09 |
| rs2028299 | *AP3S2* | C |  | 0.222 | 0.31 |  | 1.04 | 1.10 |
| rs4812829 | *HNF4A* | A |  | 0.448 | 0.29 |  | 1.05 | 1.09 |

^a^ Data from the previous report (Kooner et al. Nat Genet 43:984-989, 2011)
